# A Hint to County Hospitals

**Published:** 1907-01-12

**Authors:** 


### A HINT TO COUNTY HOSPITALS.

### POUND DAY AND ITS USES.

The committee of the Royal Surrey County Hospital are to be congratulated upon the success which has attended the institution of a Pound Day in the county of Surrey on behalf of that institution. This success appears to be due in the first instance to the skill and organising ability displayed by Mr. John Eyre, chairman, Miss M. Traill, the matron, and Mr. W. T. Patrick the assistant secretary, who were assisted by a number of ladies and gentlemen in the towns and villages of Surrey which took part in the movement. Pound Day properly organised fulfils two useful purposes. First of all it has succeeded in the county of Surrey in arousing a novel and continuous interest in the county hospital on the part of between three and four thousand people who have taken part in the work. Next it has contributed 6,453 lbs., or nearly three tons weight of various articles which have been classified under sixty-seven heads, as given below, and has brought in £54 10s. 9d. in cash. The enthusiasm excited among the poorer members of the community, especially, cannot fail to have a beneficial effect upon the county hospital. It is not an easy matter to awake enthusiasm of this kind, but where it exists the energy it represents must in a measure reach those responsible for the administration of a hospital, and so quicken them to greater efforts and increased efficiency. Every individual who becomes interested in a hospital in this way is directly and personally benefited by being brought to realise that, after all, every healthy person ought to give a little of themselves each year in the cause of the sick. We would suggest that in organising a Pound Day every hospital should issue a circular indicating the articles which would be of most use. If this could be combined with an indication of the quality of each article suggested, and where it might be obtained, the results secured must be of the best.

The Surrey County Hospital secured one or more people in each village and country town to organise the work there and distribute the circulars from house to house, mainly with the aid of the school children. In this way a very general interest is excited, and the work and claims of the county hospital can be brought in a prominent way before all classes to an extent never before accomplished. The success achieved by the Royal Surrey County Hospital is so great as to be almost, if not quite, unique in the history of Pound Days.

### What was Given.

We take the following list of articles, and the places where they came from, from our contemporary the *Surrey Advertiser* which has helped the movement considerably by the publicity generously extended to it by the editors. The local Press can do much to make a Pound Day successful. The following list containing sixty-seven articles will be examined with interest:—

| Article. | lbs. | Article. | lbs. |
|---|---|---|---|
| Tea | 591 | Flour | 235 |
| Coffee | 84 | Sweets | 21 |
| Cocoa | 86 | Maccaroni | 9 |
| Sugar | 1,220 | Semolina | 12 |
| Sago | 151 | Flannel and linen | 8 |
| Tapioca | 355 | Toys | 23 |
| Rice | 721 | Vegetables | 113 |
| Currants | 311 | Potatoes | 192 |
| Raisins | 180 | Electric lamps | 2 |
| Cornflour | 92 | Cakes | 10 |
| Biscuits | 76 | Glycerine | 1 |
| Jam | 252 | Blue | 1 |
| Honey | 22 | Note-paper | 1 |
| Marmalade | 98 | Potash | 1 |
| Pearl barley | 67 | Milk | 2 |
| Oatmeal | 18 | Linseed | 3 |
| Quaker oats | 56 | Wool and gauze | 3 |
| Soap | 27 | Mincemeat | 3 |
| Soda | 414 | Wooden leg | 3 |
| Candles | 127 | Lentils | 2 |
| Starch | 55 | Pepper | 1 |
| Butter | 12 | House flannel | 2 |
| Mustard | 12 | Curry powder | 1 |
| Cheese | 6 | Shetland wool articles | 1 |
| Bacon | 12 | Rabbits | 5 |
| Apples | 196 | Preserved ginger | 4 |
| Syrup | 47 | Groats | 10 |
| Peel | 7 | Bottle of brandy | 6 |
| Bovril, etc. | 15 | Safety pins | 1 |
| Plasmon | 6 | Bottle of disinfectant | 4 |
| Salt | 34 | Miscellaneous | 10 |
| Sultanas | 11 | Bread | 34 |
| Baking powder | 5 | | |
| Oranges | 100 | Total | 6,453 |
| Figs | 19 | | |

### Where the Pounds came from.

It is interesting to see what the individual efforts of the towns and parishes produced. The table, showing the names of the towns and villages and the amounts they contributed, is as follows:—

| | lbs. | | lbs. |
|---|---|---|---|
| Guildford | 2,893 | Runfold | 131 |
| Witley | 515 | Send | 124 |
| West Clandon | 346 | Wood Street | 124 |
| Chiddingford | 298 | Shalford | 121 |
| Farnham | 238 | Bramley | 116 |
| Pirbright | 199 | Tongham | 111 |
| Puttenham | 167 | Merrow | 106 |
| Abinger Hammer | 161 | Stoughton | 100 |
| Farnborough | 136 | Ripley | 77 |
| Milford | 132 | Godalming | 68 |

| | lbs. | | lbs. |
|---|---|---|---|
| Cobham | 62 | Leatherhead | 32 |
| Dunsfold | 50 | Shamley Green School | 15 |
| East Clandon | 45 | | |
| Shere | 45 | Total | 6,453 |
| Compton | 41 | | |

On Monday, between the hours of ten and four o'clock, the gifts were on view to the public in the Board-room at the hospital, and a large number of people availed themselves of the privilege of making an inspection.

